# Population-based mammography screening below age 50: balancing radiation-induced *vs* prevented breast cancer deaths

**DOI:** 10.1038/bjc.2011.67

**Published:** 2011-03-01

**Authors:** R de Gelder, G Draisma, E A M Heijnsdijk, H J de Koning

**Affiliations:** 1 Erasmus MC, Department of Public Health, P.O. box 2040, 3000 CA, Rotterdam, The Netherlands

**Keywords:** breast cancer, mammography, screening, radiation

## Abstract

**Introduction:**

Exposure to ionizing radiation at mammography screening may cause breast cancer. Because the radiation risk increases with lower exposure age, advancing the lower age limit may affect the balance between screening benefits and risks. The present study explores the benefit–risk ratio of screening before age 50.

**Methods:**

The benefits of biennial mammography screening, starting at various ages between 40 and 50, and continuing up to age 74 were examined using micro-simulation. In contrast with previous studies that commonly used excess relative risk models, we assessed the radiation risks using the latest BEIR-VII excess absolute rate exposure-risk model.

**Results:**

The estimated radiation risk is lower than previously assessed. At a mean glandular dose of 1.3 mGy per view that was recently measured in the Netherlands, biennial mammography screening between age 50 and 74 was predicted to induce 1.6 breast cancer deaths per 100 000 women aged 0–100 (range 1.3–6.3 extra deaths at a glandular dose of 1–5 mGy per view), against 1121 avoided deaths in this population. Advancing the lower age limit for screening to include women aged 40–74 was predicted to induce 3.7 breast cancer deaths per 100 000 women aged 0–100 (range 2.9–14.4) at biennial screening, but would also prevent 1302 deaths.

**Conclusion:**

The benefits of mammography screening between age 40 and 74 were predicted to outweigh the radiation risks.

Each year, millions of women in Europe have a screening mammogram. This may reduce the risk of dying from breast cancer by up to 50% (Allgood *et al*, 2008; Puliti *et al*, 2008; Otto *et al*, 2010). It may, on the other hand, also cause breast cancer and breast cancer deaths due to ionizing X-ray radiation. It has been shown that the risk of tumour induction is proportional to the dose of radiation absorbed in the breast (Boice *et al*, 1979; Land, 1980; Land *et al*, 1980; Howe and McLaughlin, 1996; Little *et al*, 2008; Henderson *et al*, 2010). Although radiation doses at mammography are much lower than the doses for which cancer induction is directly observed (Preston *et al*, 2002), screening a large population on a regular basis has the potential to harm.

Most nevertheless agree that the benefits of screening outweigh its radiation risks; in particular for women aged 50–69. For annual screening between age 40 and 49, the risks may also be justified, provided that a mortality reduction of at least 20% is obtained and the dose is sufficiently small (Mattsson *et al*, 2000; Berrington de Gonzalez and Reeves, 2005). The average denser breasts and faster growing tumours in this age group, however, may substantially reduce the screening effectiveness compared with older women. At the same time, the radiation risks increase with younger exposure age (Preston *et al*, 2002). Recently, the UK Age Trial showed a non-significant 24% breast cancer mortality reduction in women aged 40–48 that were annually screened (Moss *et al*, 2006). Based on these findings, we compared radiation risks to the effects of breast cancer screening, starting at various screening ages between 40 and 50 and continuing up to age 74.

For our analyses, the most recent exposure-risk model is used: the BEIR-VII model. This model differs from previous models with regard to the shape of the dose–response relation: at very low (mammography) doses, the model is adjusted with a dose and dose-rate effectiveness factor (DDREF). It is an additive instead of a multiplicative model, which until recently had been the standard model for risk estimations. We will calculate radiation risks using new estimates for the average glandular dose that were measured in the Dutch nationwide screening programme (Zoetelief *et al*, 2006), and explore the threshold for a positive benefit–risk balance.

## Materials and methods

Two models were used to estimate the ratio of screening benefits *vs* radiation risks: the micro-simulation analysis model MISCAN (de Koning *et al*, 1995; Groenewoud *et al*, 2004) that estimates the benefits of mammography screening, and the radiation risk model of the 7th Biological Effects of Ionizing Radiation committee (BEIR-VII) (National Research Council of the National Academies, 2006).

MISCAN simulates the natural course of breast cancer in the absence of screening: from its early onset to, eventually, death from breast cancer or other causes. The model also assesses the impact of screening on the natural history. In short, MISCAN is a Markov-like stage transition model, in which a lesion progresses through the successive preclinical invasive TNM stages T1a, T1b, T1c and T2+. Preclinical invasive cancer may or may not be preceded by preclinical DCIS. In the absence of screening, a lesion can grow from one preclinical stage to the next or become symptomatic. With screening, a preclinical lesion can also be screen detected. The onset rate, transition probabilities between various tumour stages, the stage durations and probability of screen detection have been estimated using observations from the Dutch cancer registry and the Dutch breast cancer screening programme (National Evaluation Team for Breast cancer screening (NETB), 2009). After a breast cancer diagnosis, some women may be cured whereas others may not. The age- and stage-specific probability of cure and the survival after diagnosis were based on several international sources (Adami *et al*, 1986; Carter *et al*, 1989; Tabar *et al*, 2000; Michaelson *et al*, 2002, 2003; Sant *et al*, 2003). The improvement in survival after screen detection was based on the Swedish breast cancer screening trials (de Koning *et al*, 1995; Tabar *et al*, 2000; Nystrom *et al*, 2002; Bjurstam *et al*, 2003). Model parameters and the procedures of parameter estimation have been published elsewhere (Groenewoud *et al*, 2004). Model outcomes were predicted breast cancer incidence and mortality without screening, and predicted breast cancer mortality with screening. From this, the predicted number of prevented deaths could be derived.

The risk of developing breast cancer due to screening exposure was estimated using the excess absolute rate (EAR) model by the BEIR-VII committee (National Research Council of the National Academies, 2006), who adopted and reparameterised the ‘pooled analysis’ EAR model by Preston *et al* (2002). The model is described as follows: 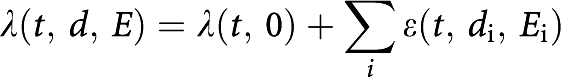 and 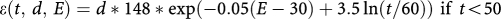


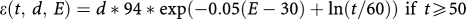


The incidence *λ*(*t*, *d*, *E*) per 100 000 women years is equal to the predicted baseline incidence without radiation *λ* (*t*, 0) plus the sum of all induced breast cancers due to radiation ∑_*i*_ *ε* (*t*, *d*_i_, *E*_i_) at each screening round. In the equation, *d* is the glandular dose (mGy), *E* is the exposure age and *t* is the attained age. The risk of radiation-induced breast cancer increases with younger exposure age and higher attained age. After age 50, the risk increases less steeply than before this age, which is probably related to hormonal changes around the menopause. The dose–response coefficient (148 before age 50, 94 from age 50) is slightly different from that in the BEIR-VII model, because an error was made in the parameterisation of the original Preston model (Preston, personal communication). The lifetime risk of breast cancer in a situation with mammography was calculated by multiplying the incidence at a given age with the survival at that age and cumulating the products: 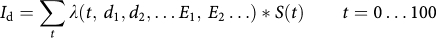


The induced breast cancer mortality was calculated by multiplying the breast cancer incidence at a given age with the survival and case fatality (*p*(*t*)) at that age, and cumulating the products for all ages: 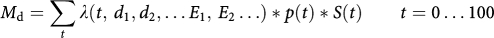


Age-specific case fatality was derived from MISCAN. Survival was calculated using a recent life table for Dutch females. We assumed that cancers that were induced by radiation could become screen detected, at a similar rate as non-induced breast cancers.

Repeated exposure to low doses such as observed at mammography screening are considered to be less harmful than the high doses that were observed in atomic bomb survivors and women that were exposed for diagnostics or therapy. Therefore, it has been suggested that the predicted number of induced breast cancers and breast cancer deaths at mammography screening should be divided by a correction factor (the DDREF) (International Commission on Radiological Protection (ICRP), 1990; National Council on Radiation Protection and Measurements (NCRP), 1993; Environmental Protection Agency (EPA), 1999; United Nations Scientific Committee on the Effects of Atomic Radiation (UNSCEAR), 2000; National Institutes of Health (NIH), 2003). In our paper, we used a DDREF of 1.5, as suggested by the BEIR-VII Committee (National Research Council of the National Academies, 2006).

Radiation risks and screening benefits were calculated for a biennial screening program targeting women aged 50–74. The impact of extending the screening programme to women younger than 50 years of age was also assessed, by calculating risks and benefits for various starting ages between age 40 and 50. The average absorbed glandular dose at mammography screening was 1.3 mGy per view (of both breasts), as shown by Zoetelief *et al* (2006) among women from four regional breast cancer screening units in the Netherlands. Two-view mammography is performed at first screening rounds, while at subsequent rounds a single view is made. Assuming that women attend all screening rounds, the total glandular dose would thus be 14 × 1.3=18.2 mGy at biennial screening between age 50 and 74. Because regional variations of between 1.04 and 1.63 mGy per view and a maximum dose of 5 mGy per view (in <1% of all women) were observed (Zoetelief *et al*, 2006), we also estimated the radiation risks for a glandular dose of 1, 2 and 5 mGy per view. For women between age 40 and 49, we assumed that the same number of views were made as for women aged 50–74. The test sensitivity was expected to be 25% lower than that of older women (International Agency for Research on Cancer, 2002). We hypothesised that breast cancer mortality could be reduced by 24% by annual mammography in screened women of this age group, similar to that observed in the UK Age Trial (Moss *et al*, 2006).

Several sensitivity analyses were performed. These include a calculation of the radiation risk:

- using the BEIR-V and BEIR-VII excess relative risk model (National Research Council, 1990; National Research Council of the National Academies, 2006),
- assuming that induced cancers could not be detected by screening,
- assuming no correction for DDREF,
- assuming a 10-year latency time,
- assuming annual screening for women aged 40–49, and biennial screening for 50- to 74-year-old women,
- assuming that the test sensitivity for women aged 40–49 is 50% lower than or similar to that for older women,
- assuming that the effectiveness of screening for women aged 40–49 is 25% lower or 25% higher than that our baseline estimate.

All effects were calculated for a cohort of 100 000 women aged 0–100, measured over their entire lifespan. To separate the consequences of radiation at younger ages from those at older ages, we also analysed the effects and radiation risks of a decade of annual screening starting at age 30 and 40. In all scenarios, a 100% participation rate was assumed. Besides screening effects, no other time-dependent changes in breast cancer incidence and mortality were assumed.

## Results

Without screening, a total of 12 289 breast cancers were predicted to be diagnosed in a population of 100 000 women aged 0–100 (Table 1). The radiation that is absorbed at biennial screening between age 50 and 74 at a glandular dose of 1.3 mGy per view would induce 7.7 extra breast cancers. The ratio of baseline incidence without screening *vs* induced incidence would be 1596 : 1, meaning that per 1596 breast cancers, 1 would be caused by screening. At a glandular dose of 5 mGy per view, the predicted number of induced breast cancers was 29.6 per 100 000 women aged 0–100, and the ratio of baseline to induced cancers 415 : 1. Extension of the lower age limit for screening would further increase the number of induced cancers, up to 17.1 if women aged 40–74 are screened biennially (1.3 mGy per view). In this scenario, the ratio baseline : induced incidence would be 720 : 1, ranging between 936 : 1 and 187 : 1 if the glandular dose would be 1 and 5 mGy per view, respectively.

The predicted number of breast cancer deaths in a situation without screening was 4330 per 100 000 women (Table 2). Approximately 26% of those (1121) could be prevented by biennial screening in the age group 50–74. On the other hand, screening would cause 1.6 extra breast cancer deaths per 100 000 women, assuming a glandular dose of 1.3 mGy per view. The ratio of baseline mortality without screening *vs* induced deaths would be 2641 : 1. Weighing the number of prevented deaths against the number of induced deaths, this ratio would be 684 : 1. A glandular dose of 5 mGy per view would increase the number of induced breast cancer deaths to 6.3, which would result in a ratio of baseline mortality *vs* induced deaths of 687 : 1, and a ratio of prevented *vs* induced deaths of 178 : 1. Screening from a younger age would further increase the number of induced deaths. Biennial screening between age 40 and 74, for instance, would cause 3.7 extra breast cancer deaths at 1.3 mGy per view. The benefit–risk ratio would then be 349 prevented *vs* 1 induced death. Increasing the glandular dose to 5 mGy per view would cause 14.4 extra breast cancers deaths, resulting in the least favourable benefit–risk ratio: 91 prevented *vs* 1 induced breast cancer death.

The balance between screening effects and radiation risks is also dependent on the assumed screening benefit for induced breast cancers, the assumed latency time and the shape of the dose–response; that is, whether the model is corrected for a DDREF or not (Table 3). Using the BEIR-V ERR model (National Research Council, 1990) instead of the BEIR-VII EAR model had no substantial effect on radiation risks, but the BEIR-VII ERR model resulted in substantially higher risk estimates. The number of induced breast cancer deaths is hardly affected by the assumed screening effectiveness or test sensitivity for women below age 50. The number of prevented breast cancer deaths, however, increases slightly, and is highest when the sensitivity is assumed to be the same for women younger and older than 50. Most breast cancer deaths can be prevented in a situation with annual screening between the ages 40 to 49, and biennial screening between age 50 and 74. However, the extra screening examinations would also result in the highest number of induced cancer deaths: 5.4 per 100 000 women. This resulted in the least favourable benefit–risk balance: per 259 prevented deaths, one breast cancer would be induced by screening.

**Figure 1:**
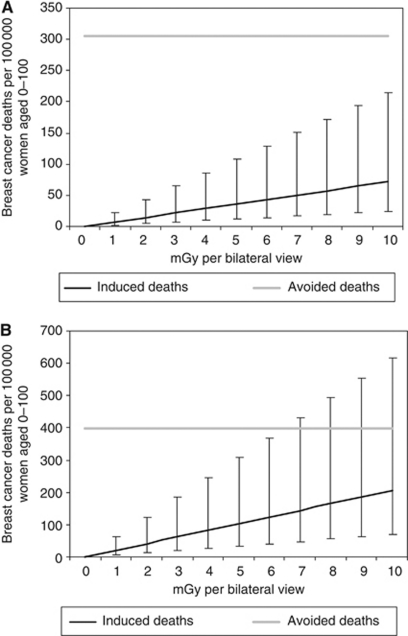
(**A** and **B**) Prevented and induced breast cancer deaths at a decade of screening starting at age 40 (**A**) or age 30 (**B**), calculated for 100 000 women aged 0–100. Calculations were based on the BEIR-VII excess absolute rate (EAR) model, assuming no latency time. The test sensitivity for women younger than 50 was assumed to be 25% lower than that for women older than 50, and the screening effectiveness was comparable to that in the UK Age Trial (Moss *et al*, 2006). For both situations, no ‘dose and dose-rate effectiveness factor’ (DDREF) correction was applied. Vertical lines represent an uncertainty interval around the estimated number of induced breast cancer deaths of a factor 3.

Focussing on the first decade of screening only, annual screening from age 40 in general would avoid more deaths than it induces. Even in the unlikely situation that the average radiation dose would exceed 10 mGy per view and the model has underestimated the number of induced breast cancer deaths by a factor of 3, the radiation risks of screening would not outweigh the benefits (Figure 1A). Annual screening from age 30 would induce more deaths than it prevents if the average dose would be 7 mGy per view or more, and the radiation risks would be underestimated by a factor of 3 (Figure 1B). However, screening in this age group in the Netherlands is only recommended when women are at high risk for breast cancer, for instance because of an inherited BRCA1 or BRCA2 gene mutation.

## Discussion

Our study demonstrated that the risk of radiation-induced breast cancer due to mammography screening is small. Biennial screening between age 50 and 74 was predicted to cause 7.7 breast cancers and 1.6 breast cancer deaths per 100 000 women aged 0–100, but would also prevent 1121 breast cancer deaths. This indicates that the radiation risks of regular mammography are negligible.

Compared with previous estimates of the radiation risk at mammography screening of 4–23 excess breast cancers (Mattsson *et al*, 2000; Law and Faulkner, 2001; Law *et al*, 2007) and 2–11 extra breast cancer deaths per 100 000 women (Beemsterboer *et al*, 1998; Mattsson *et al*, 2000; Berrington de Gonzalez and Reeves, 2005), our predictions are relatively small. This may be related to the mean glandular dose in the Netherlands of 1.3 mGy per view, which is smaller than the average dose of between 1.78 and 2.35 mGy previously found in the Netherlands (Beemsterboer *et al*, 1998), or the dose of between 1.5 and 2.4 mGy per view observed in other countries (Mattsson *et al*, 2000; Law and Faulkner, 2001; Young *et al*, 2005; Hendrick *et al*, 2010). Furthermore, calculations were based on one-view mammography at subsequent screening rounds, but in practice, two-view mammography is increasingly performed. If we would assume a second view in ∼50% of all subsequent screening rounds, as estimated by Duijm *et al* (2009), the mean glandular dose would increase to 2 mGy per examination and the number of induced breast cancer deaths to 2.5 per 100 000 women. Several screening programmes routinely use two views (Broeders *et al*, 2005), which would double the radiation risks compared with our estimates at 1.3 mGy per view.

Our estimates are also lower because a DDREF correction of 1.5 was used to estimate the radiation risk at the low doses absorbed at mammography screening (National Research Council of the National Academies, 2006). It was further assumed that all women with radiation-induced cancers could profit from the screening programme. In reality, this will not entirely be the case, because some cancers will become clinically diagnosed in the interval between two screening rounds, or after women reach the upper age limit for screening.

Radiation risk estimates involve many uncertainties. Previous calculations of the risk of mammography screening have frequently been based on an ERR model (Beemsterboer *et al*, 1998; Mattsson *et al*, 2000; Berrington de Gonzalez and Reeves, 2005), but in our study the EAR model was used, following the recommendations by BEIR-VII and Preston *et al* (2002). Our sensitivity analysis showed that the outcomes of the EAR model were comparable to those of the BEIR-V ERR model but lower than the BEIR-VII ERR model. The main conclusions did not differ. Second, different types of radiation may vary in the harm they may cause. The model that was used in this study was based on Japanese atomic bomb survivors who were exposed to *γ*-rays and neutrons, and medically exposed women who received high-energy X-rays or Ra-226 *γ*-ray radiation for diagnostics or therapy. Mammography, on the other hand, involves low-energy X-ray radiation, which may be more biologically effective, although no clear epidemiological evidence on this is currently available (National Research Council of the National Academies, 2006). The estimation of the glandular dose itself is also uncertain, and may differ by screening age, breast density and breast thickness. Regional variations in glandular dose of between 1.04 and 1.63 mGy per view have been observed in the Netherlands, which were mainly related to differences in technical conditions (i.e., anode-filter combinations) of the screening units (Zoetelief *et al*, 2006). It may be difficult to distinguish the harm due to exposure to the natural background radiation from the harm due to screening. The average natural background dose is 2.4 mSv per year (National Research Council of the National Academies, 2006), meaning that (assuming that 1 mSv=1 mGy) the cumulative glandular dose during 35 years of screening (between age 40 and 75) would be 84 mGy (2.4 × 35). As a comparison, the total screening dose would be 24.7 mGy (1.3 mGy × 18 screening rounds between age 40 and 75+1 extra view at the initial screening round).

The overall uncertainty in the assessment of radiation risks has been estimated to be a factor 2–3 (National Research Council of the National Academies, 2006; Law *et al*, 2007). Nevertheless, even if we underestimated the risks by a factor of 3, the benefits of screening for women aged 50–74 would strongly outweigh the radiation risks.

Despite the observation that radiation risks increase with younger exposure age (Preston *et al*, 2002), screening from age 40 would not severely jeopardise the benefit–risk ratio. Even if the screening effectiveness would be 25% lower and the radiation dose twice as high as in the current analysis, the radiation risks would be small. Our predictions for women between age 40 and 49 are more favourable than that of Mattsson *et al* (2000) and Berrington de Gonzalez and Reeves (2005), who expected a net increase in breast cancer deaths at a total glandular dose of ⩾50 mGy and a mortality reduction <20% (Mattsson *et al*, 2000; Berrington de Gonzalez and Reeves, 2005). The difference may be related to model choice (EAR instead of ERR). Future developments in breast cancer screening, such as digital mammography, may further increase the screening benefits for women below 50 (Pisano *et al*, 2005), and has the potential to reduce the absorbed radiation dose by 17% (Hendrick *et al*, 2010). Our results confirm that the benefit-risk ratio of screening from age 30 on is very delicate.

Of course, the risk of radiation is just one of the possible harms of mammography screening. In a decision whether or not to screen before age 50, the risks of false-positive and false-negative mammograms, as well as the risk of overdiagnosis should be taken into account.

## Conclusion

The radiation risks of mammography screening between age 40 and 74 were predicted to be negligible. From age 30, the balance between screening benefits and radiation risks would become fragile.

## Figures and Tables

**Table 1:**
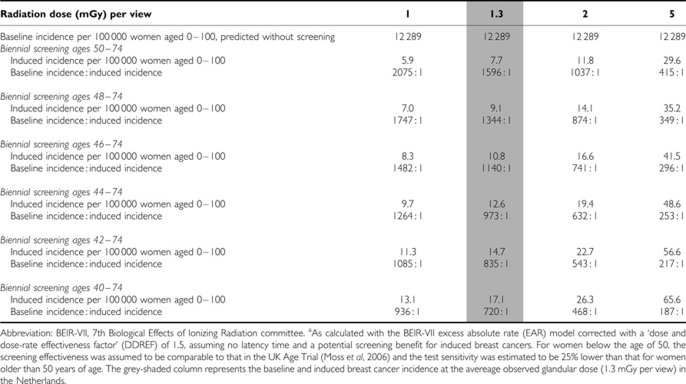
Induced breast cancer incidence at various radiation doses and screening scenarios^a^

**Table 2:**
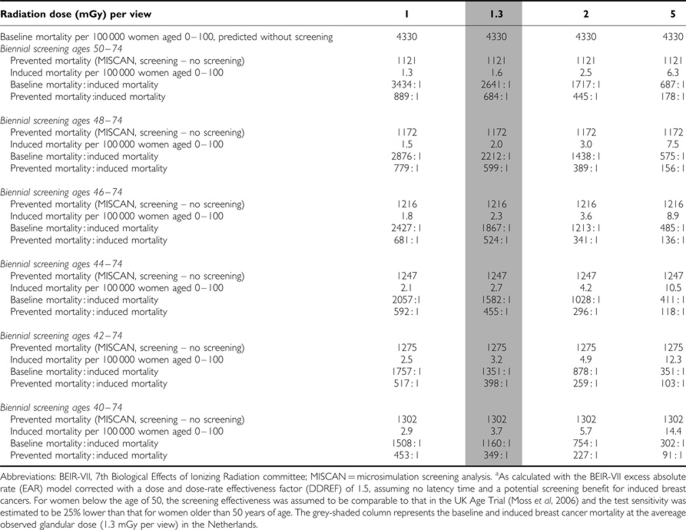
Induced breast cancer mortality at various radiation doses and screening scenarios, calculated for 100 000 women aged 0–100^a^

**Table 3:**
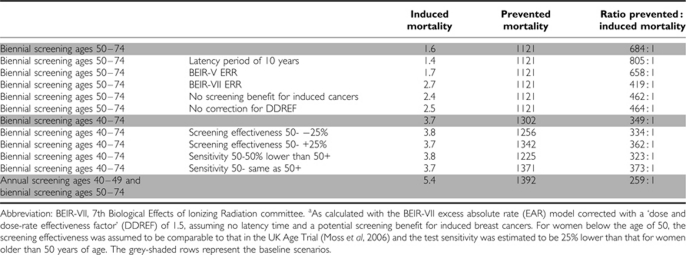
Induced and prevented breast cancer deaths and the benefit–risk ratio of breast cancer screening under various model assumptions, calculated for a glandular dose of 1.3 mGy per view, for 100 000 women aged 0–100
